# Reporter-Expressing, Replicating-Competent Recombinant Arenaviruses

**DOI:** 10.3390/v8070197

**Published:** 2016-07-20

**Authors:** Luis Martínez-Sobrido, Juan Carlos de la Torre

**Affiliations:** 1 Department of Microbiology and Immunology, University of Rochester, 601 Elmwood Avenue, Rochester, NY 14642, USA; 2 Department of Immunology and Microbial Science, The Scripps Research Institute, La Jolla, CA 92037, USA

**Keywords:** arenavirus, hemorrhagic fever, reporter genes, reverse genetic techniques, vaccines, antivirals

## Abstract

Several arenaviruses cause hemorrhagic fever (HF) disease in humans and pose an important public health problem in their endemic regions. To date, no Food and Drug Administration (FDA)-licensed vaccines are available to combat human arenavirus infections, and current anti-arenaviral drug therapy is limited to an off-label use of ribavirin that is only partially effective. The development of arenavirus reverse genetic approaches has provided investigators with a novel and powerful approach for the study of arenavirus biology including virus–host interactions underlying arenavirus induced disease. The use of cell-based minigenome systems has allowed examining the *cis*- and *trans*-acting factors involved in arenavirus replication and transcription, as well as particle assembly and budding. Likewise, it is now feasible to rescue infectious arenaviruses containing predetermined mutations in their genomes to investigate virus-host interactions and mechanisms of pathogenesis. The use of reverse genetics approaches has also allowed the generation of recombinant arenaviruses expressing additional genes of interest. These advances in arenavirus molecular genetics have also facilitated the implementation of novel screens to identify anti-arenaviral drugs, and the development of novel strategies for the generation of arenavirus live-attenuated vaccines. In this review, we will summarize the current knowledge on reporter-expressing, replicating-competent arenaviruses harboring reporter genes in different locations of the viral genome and their use for studying and understanding arenavirus biology and the identification of anti-arenaviral drugs to combat these important human pathogens.

## 1. Arenaviruses and Their Impact on Human Health

Arenaviruses cause chronic infections of rodents with a worldwide distribution [1]. Asymptomatically infected animals move freely in their natural habitat and may invade human dwellings. Humans are infected most likely through mucosal exposure to aerosols, or by direct contact between infectious materials and abrade skin. These infections are common and in some cases severe [1]. 

The family *Arenaviridae* consists currently of two genera: (1) *Mammarenavirus* and (2) *Reptarenavirus*. *Reptarenavirus* is a new genus that has been established to accommodate the distinct features of recently discovered snake arenaviruses [2,3,4,5]. Classification of the at least 25 recognized members of the genus *Mammarenavirus* into two distinct groups, Old World (OW) and New World (NW) arenaviruses [1], was originally based on serological cross-reactivity, but classification is still well supported by recent sequence-based phylogenetic studies [1]. Genetically, OW arenaviruses constitute a single lineage, while NW arenaviruses segregate into clades A, B, A/B, and C [1]. The OW arenavirus Lassa virus (LASV) is estimated to infect several hundred thousand individuals yearly in its endemic regions of West Africa, resulting in a high number of Lassa fever (LF) cases, a hemorrhagic fever (HF) disease associated with high morbidity and significant mortality [1,6,7,8,9,10]. Moreover, increased travel has led to the importation of cases of LF into the USA, Europe, Japan, and Canada [11,12,13]. Furthermore, recent studies indicating that LASV endemic regions are expanding [14] and the association of Lujo virus (LUJV) [15], a newly identified OW arenavirus, with an outbreak of HF in Southern Africa in 2008, has raised concerns about the emergence of novel HF OW arenaviruses outside their current known endemic regions. The NW arenavirus Junin virus (JUNV), endemic to the Pampas of Argentina, causes Argentine HF (AHF) with a high (15% to 30%) case-fatality rate [6] and places a population of about five million people at risk. Likewise, the NW arenaviruses Machupo virus (MACV) [16,17] and Chapare virus (CHPV) [18], Sabia virus [19,20] and Guanarito virus (GTOV) [21,22,23] are responsible for causing HF in Bolivia, Brazil and Venezuela, respectively. In addition, Whitewater Arroyo virus in the USA [24,25] and Ocozocoautla de Espinosa virus in Mexico [26] have been linked to sporadic cases of HF. Moreover, mounting evidence indicates that the worldwide-distributed prototypic arenavirus lymphocytic choriomeningitis virus (LCMV) is a neglected human pathogen of clinical significance, especially in cases of congenital infection [27,28,29,30,31]. In addition, LCMV poses a special threat to immunocompromised individuals, which has been recently illustrated by fatal cases of transplant-associated infections by LCMV [32,33,34]. OW arenaviruses LASV and LUJV and NW arenavirus JUNV, MACV, GTOV and CHPV have features that make them credible biodefense threats and have been included by the National Institute of Allergy and Infectious Diseases (NIAID) as Category A biological agents that pose a significant biodefense concern [35]. Concerns about human pathogenic arenavirus infections are aggravated by the lack of Food and Drug Administration (FDA)-licensed vaccines and antiviral drug treatment being limited to the use of ribavirin [36] that is only partially effective, associated with significant side effects and must be administrated early, and intravenously, during viral infection for optimal efficacy [37,38,39]. Evidence indicates that with morbidity and mortality of LASV, and other HF arenaviruses, infections are associated with the failure of the host’s innate immune response to restrict virus replication and to facilitate the initiation of an effective adaptive immune response [9]. Accordingly, viremia is a highly predictive factor for the outcome of LF patients [9]. Therefore, therapeutic interventions resulting in reduced virus load, without requiring virus clearance, are predicted to improve the infection outcome by promoting the recovery of appropriate host defense responses to control arenavirus multiplication and associated disease. 

Studies with the prototypic member in the family, LCMV, have led to significant advances in both virology and immunology that have been shown to universally apply to other viral infections in humans, including virus-induced immunopathological disease, major histocompatibility complex restriction and mechanisms of virus-induced immunosuppression [40,41]. The outcome of LCMV infection on its natural host, the mouse, varies dramatically depending on the host age, genetic background, strain, and immunocompetence, as well as the route of infection and the specific viral strain and dose [40,41]. This provides researchers with a unique model system to investigate parameters that influence many aspects of virus–host interactions, including the heterogeneity of phenotypic manifestations often associated with infection by the same virus.

## 2. Arenavirus Genome Organization and Virion Structure

**Figure 1:**
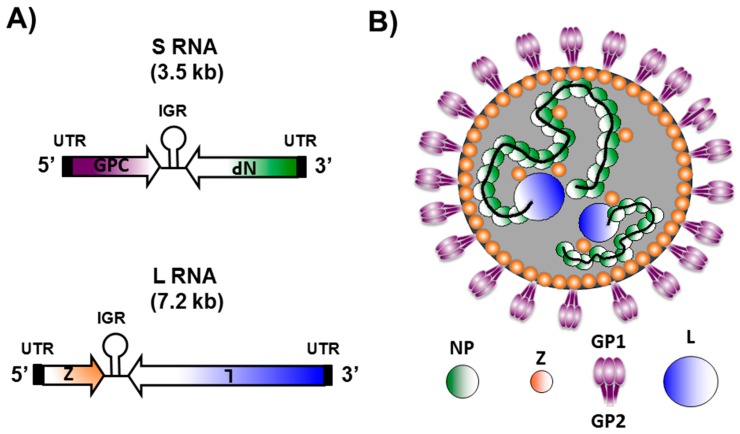
Arenavirus genome organization and virion structure. (**A**) genome organization: Arenaviruses are enveloped viruses with a single-stranded, bi-segmented RNA genome of negative polarity. Each of the two viral RNA genome segments uses an ambisense coding strategy to direct the synthesis of two viral polypeptides in opposite orientation. The Small (S) RNA segment (3.5 kb, top) encodes the viral glycoprotein precursor (GPC, purple) and nucleoprotein (NP, green). The Large (L) RNA segment (7.2 kb, bottom) encodes the RNA-dependent RNA polymerase (L, blue) and the small really interesting new gene (RING) finger protein (Z, orange); (**B**) virion structure: Arenaviruses are surrounded by a lipid bilayer containing the post-translationally processed viral glycoprotein involved in receptor binding (GP1) and viral cell entry (GP2). Underneath the lipid bilayer is a protein layer composed of the Z protein, which plays a major role in viral assembly and budding, and is the arenavirus counterpart of the matrix protein present in other enveloped negative-stranded (NS) RNA viruses. The core of the virus is made of a viral ribonucleoprotein (vRNP) complex, composed of the viral genome segments encapsidated by the viral NP. Incorporation of the vRNPs into newly nascent virions is mediated by NP-Z interaction. Associated with the vRNPs is the L polymerase protein that, together with NP, are the minimal components for viral genome replication and gene transcription.

Arenaviruses are bi-segmented negative-sense, single-stranded, RNA viruses that belong to the *Arenaviridae* family (Figure 1) [1]. Each arenaviral segment encodes, using an ambisense coding strategy, two viral proteins in opposite orientation separated by a non-coding intergenic region (IGR) (Figure 1A) [1]. The large (L) segment encodes the viral RNA dependent RNA polymerase (RdRp) or L polymerase protein (Figure 1A, blue) involved in viral replication and gene transcription [1,42]. In the opposite direction, the L segment encodes the small really interesting new gene (RING) finger protein Z (Figure 1A, orange), which is the counterpart of the matrix (M) protein present in other negative-stranded (NS) RNA viruses, and the major driving force of arenavirus assembly and budding [1,43,44,45]. The small (S) segment encodes for the viral glycoprotein precursor (GPC) (Figure 1A, purple) that is co-translationally cleaved by signal peptidase to produce a stable 58 amino acid stable signal peptide (SSP) and GPC that is post-translationally processed by the cellular Site 1 Protease (S1P) to yield the two mature virion glycoproteins (GP1 and GP2) that form the spikes that decorate the virus surface and mediate receptor recognition and cell entry [1,46,47,48]. The S segment also encodes the viral nucleoprotein (NP) (Figure 1A, green) that encapsidates the viral RNA, and together with the polymerase L and the viral RNA, constitute the viral ribonucleoproteins (vRNPs), which are the minimal factors involved in arenavirus genome replication and gene transcription [1,49,50]. In addition, arenavirus NP mediates the incorporation of the vRNPs into mature infectious virions by interacting with Z [51]. NP has also been shown to counteract the cellular host type-I interferon (IFN-I) [52,53,54,55,56,57] and inflammatory [56,58] responses during viral infection. Cellular host-derived ribosomes are also incorporated into arenavirus virions, giving them a “sandy” appearance by electron microscopy [1]. This particular property is the origin of the family name, reference to the Latin word arena (sand) [1]. However, to date, the function and relevance of the host-derived ribosomes in the viral life cycle are unknown [1]. 

## 3. Arenavirus Life Cycle

Arenavirus enveloped virions are pleomorphic but often spherical (Figure 1B). Arenavirus multiplication cycle occurs entirely in the cytoplasm of infected cells [1]. Homo-trimer complexes, consisting of the GP1 globular head and GP2 stalk region, form the spikes that decorate the surface of the arenavirus envelope [1,59] (Figure 1B). GP1, located at the top of the spike, mediates attachment of the virus particle to receptors located in the surface of the cell and is held in place by ionic interactions with the N-terminus of the transmembrane GP2 that forms the stalk of the spike [60]. Alpha-dystroglycan has been described as the main receptor for OW and NW clade C arenaviruses [61,62,63]. However, clade A, B, and A/B NW arenaviruses appear to use the human transferrin receptor protein 1 (TfR1) as the cellular receptor for viral entry [64]. Once bound to the surface of the cell, arenavirus enters the cell via receptor-mediated endocytosis [60,65]. The acidic environment of the late endosome induces a GP2 conformational change that promotes the fusion of viral and cell membranes [65], which releases the vRNPs into the cell cytoplasm where viral RNA replication and gene transcription occur [1]. 

**Figure 2:**
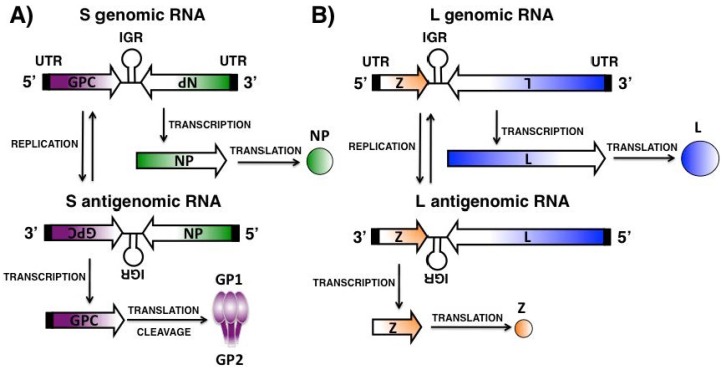
Arenavirus genome replication and gene transcription: The arenavirus replication cycle takes place entirely in the cytoplasm of infected cells. The L polymerase associated with the vRNPs initiates transcription from the viral promoter located within the untranslated region (UTR, black boxes) at the 3′ termini of the vRNAs. Primary transcription results in the synthesis of NP (**A**) and L (**B**) mRNAs from the S and L segments, respectively. Transcription termination is mediated by a secondary stem-loop structure formed by the intergenic region (IGR) found in both vRNA segments between each of the two viral genes. Subsequently, the virus polymerase L adopts a replicase mode and moves across the IGR to generate a copy of the full-length antigenome S and L vRNAs. The antigenomic RNA S and L segments serve as templates for the synthesis of GPC (**A**, S segment) and Z (**B**, L segment) mRNAs. The antigenomic RNA S and L segments also serve as templates for the amplification of the corresponding viral RNA genome species.

Arenavirus gene transcription is mediated by the viral genome and antigenome promoters located within the untranslated regions (UTRs) at the 3′ termini of viral RNA (vRNA) and complementary RNA (cRNA) species, respectively [1] (Figure 2). NP and L proteins, located at the 3′ end of the S and L viral segments, are translated from mRNAs with antigenomic sense polarity transcribed directly from the vRNAs and, therefore, are the first arenaviral proteins encoded upon infection [1] (Figure 2A,B). Transcription termination is mediated by a secondary stem-loop structure within the IGR [1]. The IGR seems to also play a role in the packaging of infectious virions [66]. GPC and Z open reading frames (ORFs) are located, correspondingly, at the 5′ end of the S and L genome segments and are translated from mRNA transcribed from the cRNAs [1] (Figure 2A,B). The cRNA species also serve as templates for the synthesis of nascent vRNAs [1]. Newly synthesized vRNAs are encapsidated by the viral NP to form the vRNP complexes and are packaged into progeny infectious virions by interaction of the viral Z [51]. Arenavirus assembly involves the interaction of the newly formed vRNP complexes with the GP1/GP2 complexes present in the membrane of infected cells, a process mediated by interaction with the Z protein [67]. Newly synthesized and assembled virions bud from infected cells, a process mediated by the Z protein [43,44,68,69]. 

## 4. Current Strategies to Combat Human Arenavirus Infections

### 4.1. Arenavirus Vaccines

The live-attenuated vaccine strain Candid#1 strain of JUNV, has been shown to be effective at combating AHF in humans without causing serious adverse effects [6,70,71]. However, outside Argentina, Candid#1 is licensed only as an investigational new drug, and studies addressing the stability of its attenuation, long-term immunity, and safety, have not been conducted. Moreover, Candid#1 does not protect against LASV [1,6,7,8,9,10]. Despite significant efforts dedicated to the development of LASV vaccines, not a single LASV vaccine candidate has entered a clinical trial. Pre-clinical work with MOPV/LASV reassortant ML29, as well as recombinant vesicular stomatitis virus (VSV) and vaccinia virus expressing LASV antigens, has shown promising results in animal models, including non-human primates, of LASV infection [72]. However, the high prevalence of HIV within LASV-endemic regions in West Africa raises safety concerns about the use of VSV- or vaccinia-based platforms. Likewise, the mechanisms of ML29 attenuation remain poorly understood, and additional mutations in ML29 could result in enhanced virulence.

The recent development of reverse genetics systems for JUNV [73,74] and LASV [75,76] could facilitate the elucidation of the genetic determinants of JUNV and LASV virulence. This, in turn, should help with the design of safer live attenuated arenavirus vaccines and thereby minimize concerns related to reversion of virulence, establishment of persistent infection and vaccination of immunocompromised individuals with live-attenuated arenavirus vaccines. 

### 4.2. Arenavirus Antiviral Drugs

In vitro and in vivo studies have documented the prophylactic and therapeutic value of the nucleoside analogue ribavirin (1-β-d-ribofuranosyl-1,2,4-triazole-3-carboxamide) against several arenaviruses [36]. Moreover, the drug has been shown to reduce significantly both morbidity and mortality associated with LASV infection in humans [36,77], and experimentally in MACV [78] and JUNV [79] infections, if given early in the course of clinical disease. The mechanisms by which ribavirin exerts its anti-arenaviral action are not fully understood and likely involve targeting different steps of the virus life cycle [80,81]. Recent evidence indicates that the nucleoside analogue can be used as a substrate by the RdRp of some riboviruses, leading to C to U and G to A transitions [82,83]. This mutagenic activity of ribavirin has been linked to its antiviral activity via lethal mutagenesis. However, the drug was also shown to strongly inhibit LCMV replication without exerting any noticeable mutagenic effect on the viral genome RNA [84]. Anemia and congenital disorders are two significant side effects associated with the use of ribavirin. In addition, oral administration is significantly less effective than intravenous administration, which poses logistic complications in regions with limited clinical infrastructure [37,38,39].

Several inhibitors of inosine-5′-monophosphate (IMP) dehydrogenase, as well as acyclic and carbocyclic adenosine analogue inhibitors of the S-adenosyl-L-homocysteine (SAH) hydrolase, have also been shown to have anti-arenavirus activity [36]. Likewise, the pyrimidine biosynthesis inhibitor A3, which exhibits broad-spectrum antiviral activity against negative- and positive-sense RNA viruses, retroviruses and DNA viruses [85], has been shown to be more efficient than ribavirin in controlling arenavirus multiplication in vitro. This inhibitory effect is due, at least in part, to its ability to interfere with viral RNA replication and transcription [86]. Moreover, since ribavirin and A3 target different metabolic pathways within the cell, they are excellent candidates for combination anti-arenaviral therapy to circumvent some limitations of monotherapy [86]. However, the antiviral effect of A3 against arenaviruses has not been evaluated in vivo. 

Various sulfated polysaccharides and phenothiazines have been reported to have activity against several arenaviruses [36]. However, in general, these compounds displayed only modest and rather non-specific effects often associated with significant toxicity. Promising results have been shown with the broad-spectrum RdRp inhibitor favipiravir, a pyrazinecarboxamide derivative, which provided protection (20% survival) in a guinea pig model of fatal AHF [87,88]. Recently, cell-based screens have identified small molecules that prevent cell entry of NW [89] and OW [90,91] arenaviruses, and whose mechanism of action appear to be based on disruption of the pH-dependent fusion event mediated by GP2. These findings illustrate how complex chemical libraries, used in the context of appropriate screening assays, can be harnessed as a powerful tool to identify candidate antiviral drugs with highly specific activities. Towards this goal, the development of arenavirus reverse genetic systems and the use of reporter-expressing, replicating competent recombinant arenavirus represent an excellent platform for the identification of novel antivirals in high-throughput screening (HTS) approaches using libraries of small molecule compounds. Moreover, recent progress in the understanding of the molecular and cell biology of arenaviruses have opened new avenues for the identification of the steps in the life cycle targeted by the identified anti-arenavirus drugs. 

### 4.3. Arenavirus Reverse Genetics

The development of reverse genetics systems to generate infectious recombinant arenaviruses from plasmid DNA has significantly advanced the investigation of arenavirus biology [1,42], including the characterization of *cis*-acting and the *trans*-acting factors that control each of the different steps of the arenavirus infectious life cycle [92,93,94,95]. Similarly, the generation of recombinant arenaviruses with predetermined mutations in their genomes has facilitated the identification and functional characterization of viral determinants of pathogenesis and associated disease in validated animal models of infection [53,96,97,98,99]. Likewise, implementation of arenavirus reverse genetics has allowed researchers to study arenavirus–host interactions [94,100,101], the role of NP in the inhibition of the IFN-I response [53,98], and the potential generation of novel live-attenuated arenavirus vaccine candidates and arenavirus-based vaccine vectors [95,100,102,103,104,105]. Advances in arenavirus molecular genetics have also led to the development of screening strategies to identify and characterize novel anti-arenavirus drugs that target specific steps of the virus life cycle [91,100]. Additionally, the use of arenavirus reverse genetics have permitted the generation of single-cycle infectious, reporter-expressing, recombinant arenaviruses, which can only replicate in GPC-expressing complementing cell lines [106,107]. These single-cycle arenavirus platforms have provided a new experimental approach to the study of some aspects of the biology of highly pathogenic arenaviruses (e.g., neutralizing antiviral responses and identification of inhibitors of GPC-mediated cell entry) without needing special biosafety conditions [106,107], which are required to study HF-causing members in the family [108]. 

Originally established for the prototyped member in the family, LCMV [109,110], plasmid-based arenavirus reverse genetics techniques have been extended to the OW LASV [75,76,111] and LUJV [112] arenaviruses, and NW JUNV [73,74], Pinchinde virus (PICV) [104,113] and MACV [114] arenaviruses. Both T7 RNA polymerase [73,75,104,109,111,113] and RNA Pol-I [74,95,100,101,102,103,110] based systems have been successfully used to launch the intracellular synthesis of the S and L RNA genome or antigenome species. These antigenome species are subsequently replicated and transcribed by the virus L and NP, the minimal viral *trans*-acting factors required for viral genome replication and gene transcription, encoded by RNA Pol-II dependent promoter protein plasmids [74,95,100,101,102,103,110]. The transcriptional activity of the RNA Pol-I exhibits species specificity, which prevents direct rescue of recombinant arenaviruses in human cells using the murine Pol-I based system [100]. This barrier was solved by the implementation of human Pol-I promoters to drive vRNA expression, which allowed for the generation of recombinant OW (LCMV) and NW (Candid#1) arenaviruses from human 293T and FDA-approved Vero cell lines [100,101]. More recently, the ability to successfully generate recombinant arenaviruses has been reduced to two plasmids by combining within the same plasmid, Pol-I-driven vRNA with Pol-II-driven protein constructs [100]. The benefit of performing arenavirus rescues using a two-plasmid approach is to increase successful co-transfection of cells that are poorly transfected, such as Vero cells, with the goal of vaccine implementation [100]. 

## 5. Reporter-Expressing Recombinant Arenaviruses

### 5.1. Recombinant Tri-Segmented (r3) Arenaviruses

**Figure 3:**
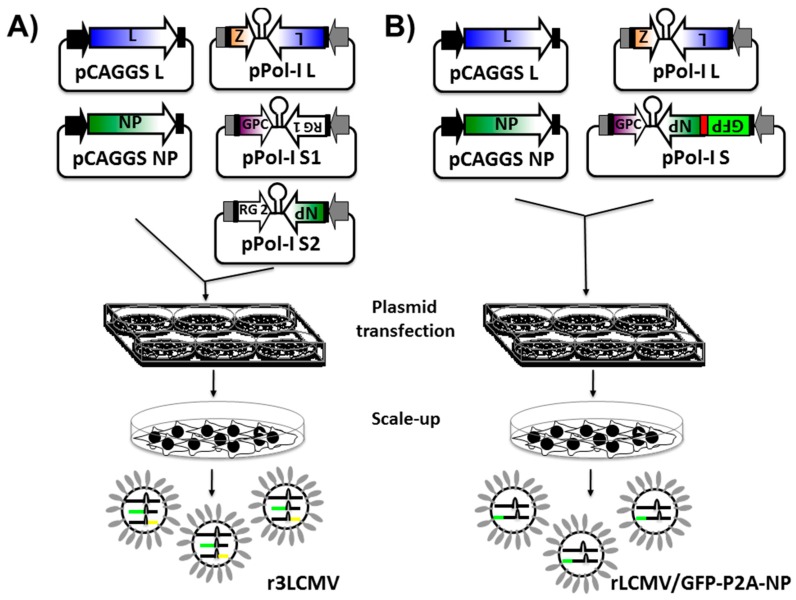
Generation of recombinant replicating competent reporter-expressing arenavirus: Arenavirus rescues are performed in rodent (using the mouse Pol-I promoter) or in human (using the human Pol-I promoter) cells in six-well plates. Alternatively, arenavirus rescues can be performed in T7-expressing cells using plasmids driving the expression of the arenaviral S and L segments under the T7 promoter. (**A**) Generation of r3 arenaviruses: Cells are transiently co-transfected, using LPF2000, with the pCAGGS protein expression plasmids encoding the viral NP and polymerase L (required to initiate viral gene transcription and genome replication) together with the pPol-I vRNA expression plasmids for the viral L segment and the two (pPol-I S1 and pPol-I S2) viral S segments. In the pPol-I S1 plasmid, the viral NP is replaced by a reporter gene 1 (RG 1), and, in the pPol-I S2 plasmid, the reporter gene 2 (RG 2) replaces the viral GPC. Alternatively, the viral NP can be replaced by RG 2 and the viral GPC by RG 1 (Figure 4A,B, respectively). At 72 h post-transfection, cells are trypsinized and scaled-up into 10 cm dishes. After an additional 72 h incubation period, presence of virus is determined by RG expression. R3 arenaviruses typically encode for two RGs, such as fluorescent or luminescent proteins (Table 1). In such cases, successful viral rescue and viral titers can be evaluated under a fluorescent microscope (i.e., fluorescent protein expression). Alternatively, a luciferase assay can be used to evaluate the presence of virus from tissue culture supernatant; (**B**) Generation of recombinant bicistronic arenaviruses: to rescue rLCMV/GFP-P2A-NP, susceptible cells are transiently co-transfected with the pCAGGS NP and L plasmids, together with the pPol-I vRNA expression plasmids encoding the L segment and the modified S segment encoding GFP-P2A-GFP (Figure 4C). At 72 h post-transfection, cells are trypsinized and scaled-up into 10 cm dishes. After an additional 72 h, presence of rLCMV/GFP-P2A-NP is determined by green fluorescent protein (GFP) expression. The chicken beta-actin promoter (black arrow) and the rabbit beta-globin polyadenylation (pA) signal (black boxes) are indicated in the pCAGGS protein expression plasmids. Viral untranslated regions (UTR, black boxes) and intergenic regions (IGR) in the pPol-I vRNA expression plasmid are indicated. The Pol-I promoter and terminator sequences in the pPol-I plasmids are indicated by gray arrows and boxes, respectively. The red box (**B**) indicates the porcine teschovirus (PTV1) 2A peptide sequence. For more details, see text.

**Figure 4:**
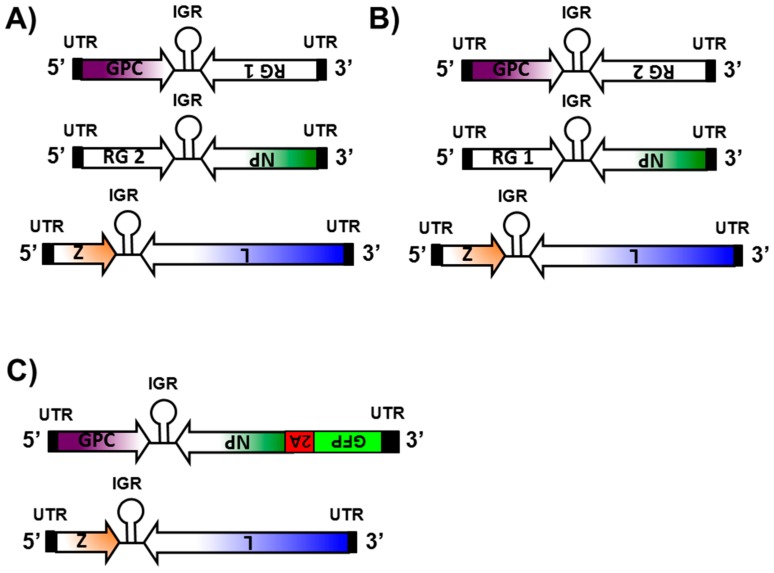
Reporter-expressing recombinant arenaviruses. (**A**,**B**) R3 arenaviruses: For the generation of r3 arenavirus, the pPol-I plasmid expressing the S vRNA segment is separated in two S plasmids. In the pPol-I S1 plasmid, the viral NP is replaced by RG 1 and in the pPol-I S2 plasmid, the viral GPC is replaced by RG 2 (**A**); alternatively, RG 1 can be expressed instead of the viral GPC in the pPol-I S2 plasmid and RG 2 from the pPol-I S1 plasmid instead of the viral NP (**B**). Regulation of RG expression depends on their location in the S segment. Expression of an RG in the NP locus is higher than that observed when the RG is located in the GPC locus. The physical separation of the GP and NP proteins into two different S segments (S1 and S2) represents a strong selective pressure to maintain a virus capable of packaging one L segment and two S segments; (**C**) recombinant bicistronic arenaviruses: In the bicistronic rLCMV/GFP-P2A-NP, the NP open reading frame in the pPol-I S plasmid is replaced by the GFP-P2A-NP sequence that contains GFP tagged to the N terminus of NP, separated by the PTV1 2A peptide sequence (P2A). The P2A sequence allows for production of both GFP and NP from the same bicistronic mRNA transcribed from the NP locus of the S genome segment. Untranslated regions (UTR, black boxes) and intergenic regions (IGR) in each of the vRNA L and S segments are indicated.

Several approaches have been used to successfully generate recombinant NS RNA viruses expressing foreign genes. These include the use of dicistronic genome segments containing internal promoters [115,116], the use of internal ribosome entry sites (IRES) [117,118], and the use of virus-specific packaging signals within vRNA segments [119,120]. Although a viable strategy in other NS segmented RNA viruses, these approaches were unsuccessful in yielding recombinant arenaviruses encoding foreign genes [103]. Successful rescue of r3 arenavirus packaging two S and one L segments into mature, infectious virions have been described for the OW arenavirus LCMV [95,100,101,103,105] and the NW arenaviruses JUNV [74,100,101] and PICV [104] (Figure 3A). Within this approach, the S segment is altered to replace one of the viral-encoded proteins (e.g., GPC and NP) by a foreign reporter gene (RG) (Figure 4A,B) [95,100,101,103,104,105]. The physical separation of the GPC and NP proteins into two different S segments (S1 and S2) represents a strong selective pressure to maintain a virus capable of packaging one L segment and two S segments [95,100,101,103,104,105]. The ability of arenaviruses to package two S segments had been suggested based on genetics [121] and structural [122,123] analysis. Moreover, because of the stability of the r3 arenaviruses, these findings suggest that production of infectious arenavirus particles containing two S and one L segments are a common event [1,103]. Importantly, each of the S segments can direct expression of a RG, and, therefore, two foreign reporter proteins can be expressed within the same virus (Figure 4A,B) [95,100,101,103,105]. Expression levels of RG are dependent on the location in the S segment [74,95,103,105]. Expression of an RG from the NP locus is greater than from the GPC locus, similar to the situation observed during viral infection (Figure 4A,B) [74,95,103,105]. Several r3 arenaviruses have been generated that express one or two additional RG [74,95,100,101,103,104,105]. Depending on the RG expressed, r3 arenaviruses showed little or no attenuation in cultured cells, and they exhibited long-term genetic stability as reflected by unaltered expression levels during serial virus passages [74,95,103,104]. In vivo, however, r3 arenaviruses exhibit significant attenuation compared to wild-type (WT) arenaviruses [103,104]. Since r3 arenaviruses are not drastically attenuated in vitro (ideal for vaccine production) but are attenuated in vivo (ideal for vaccine implementation), these r3 arenaviruses represent a great approach for arenavirus vaccine and vaccine vector development [95,100,101,104,105]. Importantly, the use of r3 arenaviruses expressing appropriate RG could be used to facilitate the identification of antiviral compounds or drugs amenable to HTS or siRNA-based library screens to identify host cell genes involved in the arenavirus life cycle [124].

To generate r3 reporter-expressing arenaviruses (Figure 3A), susceptible cells (e.g., murine or human cells using the appropriate murine or the human Pol-I promoter) [100] are co-transfected with the pCAGGS protein expression plasmids encoding NP and L, which are required to initiate viral gene transcription and genome replication (Figure 3A, left) [100,101]. The co-transfection also includes the pPol-I L segment, and two pPol-I S segments, where the GPC ORF is replaced with a RG (pPol-I S1 NP/RG 1) and the NP ORF is replaced by another RG in the second S segment (pPol-I S2 RG 2/GPC) (Figure 3A, right) [74,95,100,101,103,105]. Alternatively, the viral NP can be replaced by reporter gene one and the viral GPC by reporter gene two (Figure 4A,B) [74,95,103]. Since arenaviruses do not display classic cytopathic effect (CPE) observed with other NS RNA viruses, successful rescue of WT arenaviruses must be evaluated by performing classical plaque assays or by immunofluorescence using arenavirus-specific antibodies. Reporter-expressing r3 arenaviruses typically encode for two RG, such as fluorescent or luminescent reporter proteins [74,95,100,101,103,104,105]. In such cases, successful viral rescue and viral titers can be evaluated using fluorescent microscopy [74,95,100,101,103,104,105]. Alternatively, a luciferase assay can be used to evaluate the presence of viruses [74,95,100,101,103]. 

### 5.2. Recombinant Bicistronic Arenaviruses

The generation of a recombinant bicistronic, reporter-expressing LCMV (rLCMV/GFP-P2A-NP) has been recently described (Figure 3B) [91]. In the rLCMV/GFP-P2A-NP, the NP ORF in the pPol-I S plasmid was replaced by the bicistronic ORF GFP-P2A-NP, which contained the ORF of green fluorescent protein (GFP) tagged to the N terminus of NP and was separated by the 2A peptide (P2A) sequence derived from the porcine teschovirus (PTV1) (Figure 4C) [91]. The P2A sequence allows for production of both GFP and NP proteins from the same mRNA transcribed from the NP locus of the S genome segments (Figure 4C) [91]. Thus, GFP expression levels serve as an accurate surrogate of virus multiplication levels in infected cells [91]. To generate rLCMV/GFP-P2A-NP, rodent BHK-21 cells were co-transfected with the pCAGGS plasmids encoding the minimal components of viral replication and transcription (NP and L) (Figure 3B, left) together with the pPol-I L and the pPol-I S GFP-P2A-NP plasmids (Figure 3B, right) [91]. Because rLCMV/GFP-P2A-NP expresses GFP upon viral infection, successful virus rescue can be monitored by the presence of GFP-expressing cells [91]. Characterization of rLCMV/GFP-P2A-NP indicates that GFP expression levels were higher than those expressed from r3LCMV viruses that express GFP from either the GPC or the NP loci [91]. Importantly, the growth kinetics of rLCMV/GFP-P2A-NP in BHK-21, A549 and Vero cells, were slower early in infection but reached similar peak titers as WT rLCMV (rLCMV/WT) [91], similar to the situation observed with r3 arenaviruses [74,95,100,101,103,104]. Notably, the early growth kinetic differences between rLCMV/GFP-2A-NP and rLCMV/WT were more noticeable in human A549 cells when using a low multiplicity of infection (MOI 0.01) [91]. This probably reflects that rLCMV/GFP-P2A-NP has a modest fitness decrease that was magnified in A549 cells due to the presence of a fully active IFN-I pathway. Thus, it is possible that incomplete processing during viral infection results in a GFP-P2A-NP polyprotein that cannot counteract the IFN-I response to levels comparable to WT NP [52,53,54,55,56,57,58]. As with the r3 arenaviruses, recombinant bicistronic LCMVs expressing the GPC of LASV [48] or JUNV [125] could be used for large HTS aimed at identifying inhibitors of LASV and JUNV GPC-mediated cell entry, respectively. Whether rLCMV/GFP-P2A-NP could be used for in vivo studies remains to be determined. 

## 6. Applications of Reporter-Expressing Recombinant Arenavirus

### 6.1. Identification of Anti-Arenavirus Drugs

The development of HTS to screen a broad class of compounds that can target functions involved in different steps of the arenavirus infectious cycle would be of great value to identify potential novel anti-arenaviral drugs. This task would be facilitated by the generation of recombinant arenaviruses expressing appropriate RG, leading to the development of assays amenable to HTS. For this aim, the r3 arenavirus platform has opened the possibility of rescuing recombinant arenaviruses containing two S and one L segment(s), where each of the two S segments contains a RG instead of either GPC or NP [74,95,100,101,103,104,105]. This strategy has been used to generate a variety of r3 arenaviruses (LCMV, JUNV and PICV) expressing different reporter genes including chloramphenicol acetyltransferase (CAT), GFP and luciferases (Table 1) [74,95,100,101,103,104,105]. Notably, these r3 arenaviruses have been shown to be phenotypically and genetically very stable, providing investigators with a fantastic tool for the development of HTS to globally identify inhibitors of arenavirus multiplication [74,95,100,101,103,104,105] (Table 1). This was illustrated by the use of r3LCMV CAT/FLuc that expressed CAT and the firefly luciferase gene (FLuc) in lieu of GPC and NP, respectively, to evaluate the effect of the nucleoside analog ribavirin and DL-2-hydroxymyristic acid (2-OHM), an inhibitor of arenavirus Z myristoylation that is required for efficient viral budding, on FLuc expression and virus production [103]. Levels of FLuc activity paralleled closely to titers of infectious progeny, demonstrating that RG expression can be used as a valid surrogate of inhibition of viral infection [103]. Similarly, r3Candid#1 reporter-expressing viruses grew to high titers in cultured cells and stably expressed both reporter genes [74]. Thus, RG-expressing r3Candid#1 viruses could allow the assessment of the antiviral activity of the compounds against NW arenaviruses [74]. Accordingly, the antiviral activity of the pyrimidine biosynthesis inhibitor A3 against LCMV and JUNV (Candid#1) using r3, reporter-expressing, arenaviruses has been evaluated [86] (Table 1). Results showed that the half maximal inhibitory concentration (IC_50_) for A3 was about 100-fold lower than for ribavirin [86]. The anti-arenaviral activity of A3 was also observed in different cell types and species, including human A549 cells, and at drug concentrations that showed minimal effects on cell viability [86]. Moreover, readouts based on RG expression levels and viral titers gave similar IC_50_ values for each compound, similar to findings obtained with rLCMV/WT and rCandid#1/WT, further validating the use of r3 arenaviruses to screen for antiviral compounds using RG expression as readouts [86]. The antiviral activity of A3 on arenavirus was reverted by the exogenous addition of orotic acid, suggesting the involvement of the de novo pyrimidine biosynthesis pathway as the primary target of A3 [86]. Ribavirin and A3 appear to target different processes involved in arenavirus multiplication, and accordingly their use in combination therapy exhibited more potent arenavirus inhibitory activity than either single-drug treatment [86]. This combinatory therapy would allow circumventing some of the limitations of the current ribavirin monotherapy used for the treatment of arenavirus infection [1]. The recombinant bicistronic rLCMV/GFP-P2A-GFP has been also used for the identification of inhibitors of LCMV infection in the context of a cell-based HTS format (Table 1) [91]. Altogether, these results demonstrate the feasibility of using reporter-expressing r3 or bicistronic arenaviruses in cell-based assays to identify compounds with antiviral activity. Moreover, since RG expression can be used as a valid surrogate for viral replication, the replicating competent, RG-expressing arenaviruses can be used to screen large libraries of compounds for the rapid identification of compounds with anti-arenaviral activity [91].

### 6.2. Studying the Biology of Arenaviruses In Vitro

Evidence indicated that r3LCMVs expressing different RG were stable both genetically and phenotypically and exhibited rLCMV/WT-like growth properties in culture cells [103]. Accordingly, RG expression from r3LCMV-infected cells provided an accurate surrogate of virus multiplication levels [86,103]. These results demonstrate the feasibility of using r3LCMVs to study several aspects in the biology of arenavirus in cell culture [86,103]. Thus, r3LCMV CAT/GFP (where CAT substituted for the viral GPC and GFP substituted for the viral NP) and r3LCMV GFP/CAT (where GFP substitute for the viral GPC and CAT substituted for the viral NP) (Table 1) (Figure 4A,B) [103] had similar growth properties in cultured cells, but normalized levels of CAT activity or GFP expression revealed higher levels of expression when the reporter gene was in the NP loci rather than in the GPC loci [103]. These results were further confirmed by results with rLCMV containing rearranged ORFs as a new approach to develop live attenuated vaccines [95], as well as with different r3LCMV expressing GFP or Gaussia luciferase (Gluc) from the NP or GPC loci (Table 1) [95]. Importantly, differences in RG expression by the different r3LCMV were not due to differences in numbers of infected cells or viral growth kinetics [95]. Consistent with these results, Cheng et al. generated a rLCMV containing a translocated viral S segment (rLCMV/TransS) where the viral NP and GPC ORF replaced one another [95]. The rLCMV/TransS showed slower growth kinetics in cell culture and was completely attenuated in a mouse model of lethal LCMV infection [95]. Notably, a single immunization dose of rLCMV/TransS conferred complete protection against a lethal challenge with rLCMV/WT [95]. To gain insights and to demonstrate that the mechanism of attenuation for LCMV/TransS was associated with reduced NP expression levels, Cheng et al. generated r3LCMVs containing both the NP and GPC genes under the regulation of either the antigenome (r3LCMV/TransNP) or the genome (r3LCMV/TransGPC) promoters [95]. To prevent discrepancies in measurements of RG expression under conditions of different UTRs, Cheng et al. used for comparison r3LCMVs encoding GFP and Gluc in positions corresponding to those found in r3LCMV/TransNP and r3LCMV/TransGPC (Table 1) [95]. They observed that r3LCMV/TransNP replicated similarly to r3LCMV/TransS. Moreover, viral growth kinetics corroborated that r3LCMV/TransS and r3LCMV/TransNP exhibited similar degrees of attenuation in cell culture [95]. In contrast, r3LCMV/TransGPC-infected and r3LCMV/WT-infected cells expressed similar GFP levels and viral growth kinetics, demonstrating that the two viruses also had similar replication capabilities [95]. Together, these results demonstrated that reduced NP expression levels, rather than increased GPC expression levels, were the major contributor of the impaired growth properties, and probably in vivo attenuation, observed with rLCMV/TransS [95].

In addition to demonstrating regulation of NP and GPC expression, results using r3LCMVs suggested that arenavirus coding regions do not seem to play a role in viral packaging and, therefore, both GPC and NP could be entirely replaced by a foreign gene without any attenuation in cell culture [74,86,95,100,101,103,104]. It remains to be evaluated the total length of a foreign RG to be incorporated and rescued in a r3 arenavirus. To date, FLuc (1.6 kb) is the largest foreign gene rescued in a r3LCMV (r3LCMV FLuc/FLuc) with a total genome size of 14.1 kb, compared to the 10.6 kb of r3LCMV/WT (Table 1) [103]. Notably, r3LCMV FLuc/FLuc was attenuated both in vitro and in vivo, suggesting a limit in the length of foreign genes that can be inserted in r3LCMVs [103]. Thus, the r3 arenavirus approach not only offers an opportunity to study the mechanisms responsible for controlling virus gene expression but also genome packaging for efficient viral fitness [103]. Moreover, the confirmation that arenavirus virions can package two S and one L genome segments in the r3 arenaviruses suggest this could be a very common event during the replication cycle of arenaviruses [121,122,123]. Likewise, it remains to be determined the size limit of the RG that can be expressed using the bicistronic approach without significantly affecting viral fitness [91].

### 6.3. Reporter-Expressing Arenaviruses for In Vivo Studies

Tri-segmented arenaviruses expressing RG provide investigators with a novel tool for the investigation of virus–host interactions in vivo. However, in contrast to results observed in cell culture, compared to rLCMV/WT, the r3LCMV GFP/GFP was attenuated in a mouse model of fatal lymphocytic choriomeningitis (LCM) following intracranial (i.c.) virus inoculation, as reflected by lower and delayed mortality [103]. By day 8 post-infection, only 37.5% of r3LCMV GFP/GFP-infected mice died and the remaining symptomatic mice recovered and did not show noticeable clinical symptoms by day 9 post-infection [103]. Nevertheless, the use of a higher i.c. dose (10^5^ focus forming units, FFU) of r3LCMV GFP/GFP resulted in 100% lethality by day 7 post-infection [103]. Thus, although attenuated in mice, r3LCMVs can still be used for in vivo studies by increasing the amount of virus needed to obtain a phenotype similar to that of rLCMV/WT [103]. This outcome is similar to the situation observed with other replicating competent, RG-expressing NS segmented viruses, such as influenza [126,127]. To evaluate if a protective immune response was established in mice that survived the i.c. inoculation with 10^3^ FFU of r3LCMV GFP/GFP, animals were challenged at day 21 post-infection with 10^3^ FFU of rLCMV/WT [103]. All r3LCMV GFP/GFP inoculated mice exhibited complete protection [103]. The r3LCMV GFP/GFP and rLCMV/WT exhibited the same tropism within the brain of infected mice, although r3LCMV GFP/GFP displayed a more restricted distribution [103]. Cells in the ependymal, choroid plexus, and olfactory bulb were clearly infected by r3LCMV GFP/GFP, whereas infection of the meninges was patchy compared with rLCMV/WT [103]. GFP expression in r3LCMV GFP/GFP-infected mice was of sufficient intensity to be detected without the need for amplification or secondary quantification approaches, demonstrating the feasibility of using r3LCMV GFP/GFP for in vivo studies to identify the presence of infected cells [103]. 

The r3LCMV technology has also been used to express interleukin-10 (IL-10) and Cre recombinase genes in vivo (Table 1) [105]. Mice infected (i.c., 10^4^ FFU) with r3LCMV/GFP-IL-10, but not r3LCMV GFP/GFP, were protected from lethal LCM, probably because IL-10 expression during viral infection leads to a decrease in immunopathology due to reduced CTL activity and modulation of macrophages and neutrophils pro-inflammatory activities [105]. These results support the potential of using r3LCMVs to determine the biological effects of candidate immune molecules, or other host genes, during the natural course of LCMV infection in vivo [105]. Infection of IL-10-deficient (IL-10−/−) mice with r3LCMV GFP/IL-10 resulted in a large number of hybridomas producing antibodies to IL-10 after a single immunization that after boosting resulted in several high-affinity clones specific to IL-10 [105]. These results also suggest the potential of using r3LCM viruses to generate antibodies, where LCMV infection can act as a natural adjuvant [105]. 

The characterization of a recombinant Pichinde virus, PICV (strain P18) with a trisegmented RNA genome (rP18tri-G) that expresses GFP from the GPC locus (Table 1) [104], showed that, similarly to other documented tri-segmented arenaviruses, the rP18tri was attenuated compared to rPICV/WT, but exhibited stability during serial passages in cultured cells [104]. rP18tri viruses expressing GFP from the GPC locus and either the hemagglutinin (HA, rP18tri-G/H) or the nucleoprotein (NP, rP18tri-G/P) of influenza A/Puerto Rico/8/34 (PR8) H1N1 from the NP locus have been also generated (Table 1) [104]. Mice immunized with a single low dose of rP18tri-G/H were protected against a lethal challenge with influenza PR8 [104]. Moreover, rP18tri-G/H was able to efficiently induce high levels of neutralizing antibodies against PR8 HA using intramuscular (i.m.), intranasal (i.n.) or intraperitoneal (i.p.) routes of infection [104]. Furthermore, the antibody neutralization titers were comparable to those induced by a formalin-inactivated influenza PR8 virus [104]. Likewise, mice immunized with rP18tri-G/P generated virus-specific CD8 and CD4 T cell responses above the background level seen in the rP18tri-G control group [104]. These data demonstrate that the rP18tri viruses can efficiently induce both humoral and cell-mediated immune responses via different immunization routes, leading to efficient protection. These results further demonstrate the feasibility of using the r3 arenavirus approach as a novel vaccine platform [104]. 

## 7. Conclusions

The development of arenavirus reverse genetics systems has provided investigators with a novel and powerful experimental approach to study basic aspects of arenavirus biology, including the identification of viral determinants, and their mechanisms of action, which contribute to arenaviral human diseases. The ability to manipulate the genome of arenaviruses has proven to be a superb model system to study virus–host interactions and associated disease. Moreover, generating recombinant arenaviruses with predetermined mutations allows investigators to gain a detailed understanding of arenavirus–host interactions and phenotypic outcomes of virus infection. Furthermore, the generation of r3 or bicistronic arenaviruses expressing appropriate RG, together with the development of specific cell-based assays for each of the different steps of the arenavirus life cycle, is facilitating novel approaches to discover and characterize antiviral drugs against these important human pathogens. 

## Figures and Tables

**Table 1:** Reporter-expressing recombinant arenaviruses.

Virus	NP Loci	GPC Loci	Reference
r3LCMV GFP/CAT	CAT	GFP	[103]
r3LCMV CAT/GFP	GFP	CAT	[86,103]
r3LCMV GFP/GFP	GFP	GFP	[103,105]
r3LCMV CAT/FLuc	FLuc	CAT	[103]
r3LCMV FLuc/FLuc	FLuc	FLuc	[103]
r3LCMV GFP/Gluc	GFP	Gluc	[86,95,100,101]
r3LCMV Gluc/GFP	NP	GFP	[95]
r3LCMV/TransS GFP/Gluc	Gluc	GFP	[95]
r3LCMV/TransS Gluc/GFP	GFP	Gluc	[95]
r3LCMV/TransS GFP/Gluc	GFP	Gluc	[95]
r3LCMV GFP/IL-10	IL-10	GFP	[105]
r3LCMV GFP/Cre	Cre	GFP	[105]
rLCMV/GFP-P2A-NP	GFP	GPC	[91]
r3Candid#1 GFP/CAT	GFP	CAT	[74,86]
r3Candid#1 CAT/GFP	CAT	GFP	[74]
r3Candid#1 GFP/Gluc	GFP	Gluc	[86,101]
r3PICV GFP (rP18tri-G)	No RG	GFP	[104]
r3PICV GFP (rP18tri-G/H)	Influenza HA	GFP	[104]
r3PICV GFP (rP18tri-G/P)	Influenza NP	GFP	[104]

GFP: green fluorescent protein; r3: recombinant tri-segmented; LCMV: lymphocytic choriomeningitis virus; CAT: chloramphenicol acetyltransferase; FLuc: firefly luciferase; Gluc: Gaussia luciferase; PICV: Pinchinde virus; RG: reporter gene; HA: hemagglutinin; NP: nucleoprotein; IL-10: interleukin-10.
